# Functional Mitochondria in Health and Disease

**DOI:** 10.3389/fendo.2017.00296

**Published:** 2017-11-03

**Authors:** Patries M. Herst, Matthew R. Rowe, Georgia M. Carson, Michael V. Berridge

**Affiliations:** ^1^Cancer Cell Biology, Malaghan Institute of Medical Research, Wellington, New Zealand; ^2^Department of Radiation Therapy, University of Otago, Wellington, New Zealand; ^3^School of Biological Sciences, Victoria University, Wellington, New Zealand

**Keywords:** mitochondrial DNA, mitochondrial DNA mutations, mitochondriopathies, mito-nuclear cross talk, mitochondrial transfer, mitochondrial stress signals, mitopeptides

## Abstract

The ability to rapidly adapt cellular bioenergetic capabilities to meet rapidly changing environmental conditions is mandatory for normal cellular function and for cancer progression. Any loss of this adaptive response has the potential to compromise cellular function and render the cell more susceptible to external stressors such as oxidative stress, radiation, chemotherapeutic drugs, and hypoxia. Mitochondria play a vital role in bioenergetic and biosynthetic pathways and can rapidly adjust to meet the metabolic needs of the cell. Increased demand is met by mitochondrial biogenesis and fusion of individual mitochondria into dynamic networks, whereas a decrease in demand results in the removal of superfluous mitochondria through fission and mitophagy. Effective communication between nucleus and mitochondria (mito-nuclear cross talk), involving the generation of different mitochondrial stress signals as well as the nuclear stress response pathways to deal with these stressors, maintains bioenergetic homeostasis under most conditions. However, when mitochondrial DNA (mtDNA) mutations accumulate and mito-nuclear cross talk falters, mitochondria fail to deliver critical functional outputs. Mutations in mtDNA have been implicated in neuromuscular and neurodegenerative mitochondriopathies and complex diseases such as diabetes, cardiovascular diseases, gastrointestinal disorders, skin disorders, aging, and cancer. In some cases, drastic measures such as acquisition of new mitochondria from donor cells occurs to ensure cell survival. This review starts with a brief discussion of the evolutionary origin of mitochondria and summarizes how mutations in mtDNA lead to mitochondriopathies and other degenerative diseases. Mito-nuclear cross talk, including various stress signals generated by mitochondria and corresponding stress response pathways activated by the nucleus are summarized. We also introduce and discuss a small family of recently discovered hormone-like mitopeptides that modulate body metabolism. Under conditions of severe mitochondrial stress, mitochondria have been shown to traffic between cells, replacing mitochondria in cells with damaged and malfunctional mtDNA. Understanding the processes involved in cellular bioenergetics and metabolic adaptation has the potential to generate new knowledge that will lead to improved treatment of many of the metabolic, degenerative, and age-related inflammatory diseases that characterize modern societies.

## Introduction

**Figure 1:**
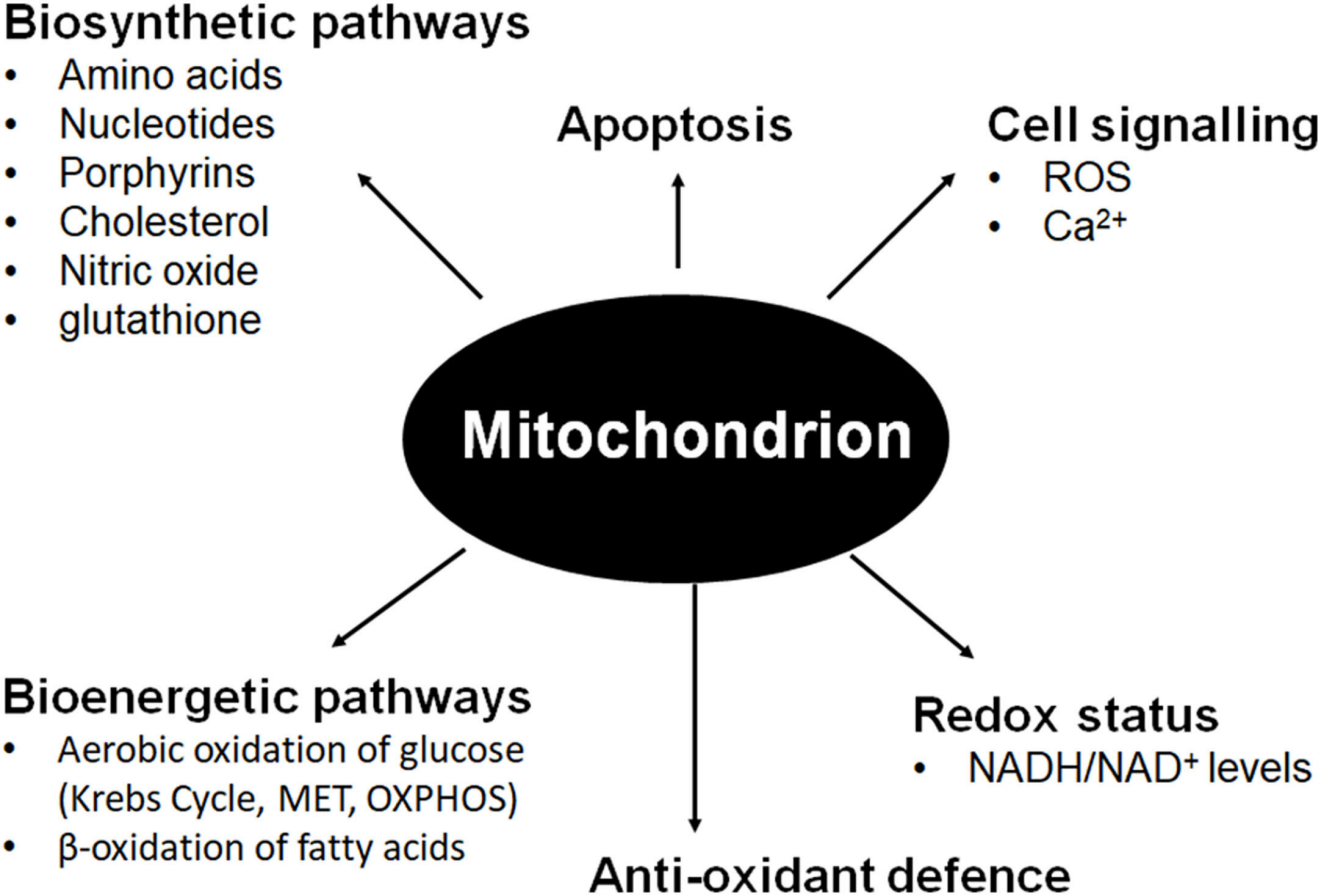
Mitochondrial involvement in fundamental cellular pathways and processes.

Mitochondria are maternally inherited multifunctional organelles that form a comprehensive network in many cells maintained by an intricate balance between fission and fusion, mitochondrial biogenesis, and mitophagy (1, 2). Although mitochondria are best known for harvesting and storing energy released by the oxidation of organic substrates under aerobic conditions through respiration, their many anabolic functions are often overlooked (see Figure 1). Arguably, the biosynthetic functions of mitochondria are at least as important for tumorigenesis and tumor progression as ATP generation [recently reviewed by Ahn and Metallo (3)]. Tumor cells easily survive in hypoxic conditions by recycling NADH to NAD^+^ *via* lactate dehydrogenase (LDH) and plasma membrane electron transport (PMET) to allow for continued glycolytic ATP production (4). Cells without mitochondrial (mt) DNA (ρ^0^ cells) are incapable of mitochondrial electron transport (MET) coupled to oxidative phosphorylation (OXPHOS), but proliferate if supplemented with pyruvate and uridine (5, 6). Pyruvate addition appears to be necessary to maintain the pyruvate/lactate couple which generates NAD^+^ for continued glycolysis, even though the majority of pyruvate produced through glycolysis will be reduced to lactate rather than entering the Krebs cycle, which limits biosynthetic intermediates required for several metabolic pathways (3, 5). For example, α-ketoglutarate is a precursor of glutamate, glutamine, proline, and arginine while oxaloacetate produces lysine, asparagine, methionine, threonine, and isoleucine. Amino acids in turn are precursors for other bioactive molecules, such as nucleotides, nitric oxide, glutathione, and porphyrins. Citrate can be transported out of mitochondria *via* the pyruvate-citrate shuttle and metabolized to cytosolic acetyl-CoA, which is the substrate for the biosynthesis of fatty acids and cholesterol as well as protein acetylation (3). Uridine is necessary for ρ^0^ cells to bypass metabolic reliance on MET, allowing continued pyrimidine biosynthesis and thus DNA replication to continue. Dihydroorotate dehydrogenase (DHODH), a flavoprotein found on the outer surface of the inner mitochondrial membrane (IMM), oxidizes dihydroorotate to orotate. Electrons from this oxidation are used to reduce coenzyme Q just prior to complex III in MET (6). In the absence of MET, DHODH is unable to oxidize dihydroorotate, blocking pyrimidine biosynthesis.

Whereas many biosynthetic processes occur in the mitochondrial matrix, respiratory complexes that form the functional respirasome are positioned in the IMM, which is heavily folded into cristae in many cell types with high energy requirements. Electrons from NADH and FADH^2^ are transported to oxygen as the terminal electron acceptor through respiratory complexes I, II, III, and IV of MET. The energy released in this process is stored in the form of a proton gradient, which produces an electric potential across the IMM. This membrane potential drives the generation of ATP through OXPHOS *via* the F_0_F_1_ ATP synthase (respiratory complex V) [summarized in Ref. (7)]. The mitochondrial membrane potential also regulates influx of Ca^2+^ ions into the mitochondria to buffer cytoplasmic calcium as well as facilitate the import of nuclear-encoded, mitochondrially targeted proteins (n-mitoproteins) (7–10). MET ensures low NADH/NAD^+^ ratios to facilitate sustained glycolysis. An important byproduct of MET is the production of reactive oxygen species (ROS) which at low levels act in cell signaling pathways. These radicals are balanced by strong mitochondrial antioxidant defense systems to prevent oxidative damage to mitochondrial DNA (mtDNA), and to protein and lipids at higher concentrations (11, 12). Mitochondria are also involved in regulation of apoptosis through activation of the mitochondrial permeability transition pore whenever ROS and the AMP/ATP ratio increases and Ca^2+^ levels in the mitochondria increase (13, 14).

Mitochondria play a vital role in bioenergetic and biosynthetic pathways and can rapidly adapt to meet the metabolic needs of the cell. Increased demand is met by mitochondrial biogenesis and fusion of individual mitochondria into dynamic networks, whereas a decrease in demand results in the removal of superfluous mitochondria though fission and mitophagy (1, 2, 15, 16). This level of adaptability to cellular needs is achieved by effective communication between the nucleus and the mitochondria. Factors that compromise mito-nuclear cross talk will affect the cell’s ability to respond to stresses caused by changes in the microenvironment. Effective mito-nuclear cross talk is also of vital importance in tumorigenesis and tumor progression but remains largely unexplored (17). The role of mitochondria in different stages of tumor biology has been reviewed recently (10, 18–20). This review will discuss how cells respond when mtDNA mutations accumulate, mito-nuclear cross talk falters, and mitochondria do not deliver important functional outputs. In some cases, drastic measures such as acquisition of new mitochondria from donor cells occur to ensure cell survival.

## Evolutionary Origin of Mitochondria

To better understand the need for and intricacies of ongoing mito-nuclear communication, we provide a brief summary of the events that led to mitochondria becoming an integral part of eukaryotic cells. The idea that mitochondria originated from free living bacteria that became incorporated inside an archaeal cell *via* endocytosis (21) is supported by phylogenetic analysis of conserved ribosomal RNA (rRNA) (22, 23). It is now widely accepted that all multicellular life originated from a common eukaryotic ancestor that evolved more than two billion years ago. Although the exact timing of the acquisition of the α-proteobacterium is still debated (24, 25), the metabolic advantages that this endosymbiotic relationship brought are indisputable. Protomitochondria conferred on early eukaryotes the ability to use previously toxic oxygen to fuel a much more efficient way of releasing energy from organic substrates, aerobic respiration. This allowed them to colonize new and diverse ecological niches and set the scene for the advent of complex multicellularity, eventually giving rise to fungal, plant, and animal cells. A comparison of non-ribosomal proteomes of α-proteobacteria and eukaryotes reveals that protomitochondrial metabolism was likely based on the aerobic catabolism of lipids, glycerol, and amino acids provided by the host. Over time, considerable endosymbiotic gene transfer from the protomitochondrion to the host nucleus occurred while many genes were lost through redundancy. As a result, the nuclear genome has become larger and more complex, while the mitochondrial genome has dwindled. A comparison of proteomes suggests that only 22% of human mitochondrial proteins are of protomitochondrial descent (26).

## Integrity of mtDNA

### Organization of mtDNA in Nucleoids

The human mitochondrial genome is a double-stranded, closed-circular molecule of 16,569 nucleotide pairs. It was first sequenced in 1981 (27) and revised in 1999 (28). mtDNA does not contain introns and encodes just 13 polypeptides, 22 transfer RNAs (tRNAs), and the 12S and 16S rRNA genes for mitochondrial protein synthesis (29). The 13 polypeptides encode subunits of the respiratory complexes (7 of 45 for RC-I, 1 of 11 for RC-III, 3 of 13 for RC-IV, and 2 of 16 for RC-V). The four subunits that make up RC-II are nuclear encoded along with the remaining 85% of the other RC subunits (29). Nuclear DNA encodes more than 22,000 proteins, about 1,500 of which contribute to the mitochondrial proteome. These n-mitoproteins include enzymes required for the TCA cycle, amino acid, nucleic acid and lipid biosynthesis, mtDNA and RNA polymerases, transcription factors, ribosomal proteins in addition to all components of DNA repair pathways. N-mitoproteins are expressed in the cytoplasm and folded upon entry through the mitochondrial outer membrane *via* the TOM/TIM complex. From there, they locate to their specific sites: the outer mitochondrial membrane (OMM), the IMM, the intermembrane space (IMS), or the mitochondrial matrix (30).

Mitochondrial DNA is not structurally associated with histones, as is nuclear DNA. Instead, it is closely associated with a number of proteins in discreet nucleoids, approximately 100 nm in diameter. Nucleoids are anchored to the IMM, facing the matrix (31). One cell can contain tens to thousands of nucleoids, each with a single mtDNA molecule as shown by super-resolution microscopy (31). Transcription factor A of mitochondria (TFAM), the mtDNA helicase Twinkle, and mitochondrial single-stranded DNA-binding protein (mtSSB) all co-localize with mtDNA within nucleoids (32). TFAM binds to mtDNA and forces U-turns in the circular molecule, which allows compacting and packaging of mtDNA into nucleoids (33). TFAM plays an important role in both the transcription and maintenance of mtDNA and has been shown to recognize and bind to cisplatin-damaged and oxidized mtDNA. TFAM is also expressed in the nucleus and regulates nuclear genes. mtDNA replication is enhanced by an increase in expression of the master regulator of the mitochondrial biogenesis, transcriptional coactivator peroxisome proliferator-activated receptor gamma coactivator 1alpha (PGC-1α) expression *via* co-activation of the nuclear respiratory factor 2 (NF2), and NF1 (34). Overexpression of mitochondrial TFAM after cisplatin exposure promotes treatment resistance and cancer growth (35). High TFAM expression in tumors has been found to be correlated with poor outcomes in patients with ovarian cancer, pancreatic adenocarcinoma, endometrial adenocarcinoma, and colorectal cancer, with a poor response to chemotherapy [reviewed by Kohno et al. (35)]. TFAM, Twinkle, and mtSSB are essential components of nucleoids. Other n-mitoproteins associated with mtDNA replication, transcription, translation, and repair are transiently associated with the nucleoid and are referred to as mitochondrial nucleoid associated proteins (30). Mito-ribosomes, although found close to nucleoids, are not attached and are, therefore, not part of the nucleoid. In contrast to prokaryotes, mitochondrial transcription and translation occur as separate processes, with the polycistronic RNA needing further processing before being translated. Any apparent association with the nucleoid is likely to be related to the small spaces between cristae in the mitochondrial matrix (30).

The mitochondrial and nuclear genomes differ in size by more than five orders of magnitude. However, each somatic cell contains 10 to several thousand mtDNA copies (31) and only two copies of nDNA. This disproportionate representation of protein-encoding mitochondrial to nuclear genes in most cells exceeds two orders of magnitude (36), requiring ongoing mito-nuclear communication to ensure appropriate stoichiometry of RC subunits. Both decreased and increased mtDNA copy numbers have been associated with increased cancer incidence, with contradictory findings between some studies for the same type of tumor. For example, both increased (37) and decreased copy numbers (38, 39) have been reported to increase the incidence of renal cancer.

Given the density of open reading frames in mtDNA, one could argue that loss of mtDNA integrity can have serious consequences for individual cells as well as the entire organism. It is generally accepted that mtDNA mutates more rapidly than nDNA because of its close proximity to mitochondrially generated ROS, lack of protective histone proteins, and comparatively less effective repair processes (40, 41). An earlier study showed that mtDNA damage is not only more severe but also persists longer than nuclear DNA damage after H_2_O_2_ exposure (42). However, other studies have shown that the DNA-binding proteins of mitochondrial nucleoids can be equally protective of mtDNA as histones are of nuclear DNA when exposed to H_2_O_2_ or X-rays (43). In addition, the effect of a single mtDNA mutation may have fewer consequences than a single nDNA mutation. Impaired MET due to mtDNA mutations results in depolarized mitochondria which are unable to re-fuse with the mitochondrial network after fission (44). Protection from damage by nucleoid proteins combined with the removal of mitochondria with damaged DNA through mitophagy may result in a more robust response to oxidative stressors such as H_2_O_2_ than previously thought.

Cells of most outbred populations contain more than one mitochondrial genotype. This heteroplasmy can be quite variable within tissues and cell types of one organism, complicating interpretation of mitochondrial genetics and influencing disease presentation in the case of pathological mtDNA mutations (36). Interestingly, most mtDNA mutations are recessive and easily complemented by wild type mtDNA copies. There seems to be a threshold ratio of mutated/wild type mtDNA of approximately 70% before disease symptoms become evident, depending on the mutation and the type of tissue (40, 45).

Both nDNA mutations that affect n-mitoproteins and mtDNA mutations in the 13 genes encoding subunits of the respiratory chain compromise OXPHOS (40, 41, 45). Germline mutations, resulting in a decrease in or loss of expression of succinate dehydrogenase (SDH), fumarate hydratase (FH), and isocitrate dehydrogenase have been reported in inherited paragangliomas, gastrointestinal stromal tumors, pheochromocytomas, myomas, SDH, papillary renal cell cancer (FH), and gliomas (46).

Mutations in mtDNA have been implicated in neuromuscular and neurodegenerative mitochondriopathies (47–49) and complex diseases like diabetes (50), cardiovascular diseases (51, 52), gastrointestinal disorders (53), skin disorders (54), aging (55, 56), and cancer (41).

A recent review by van Gisbergen et al. (41) describes several studies showing that mtDNA germline variations can play a role in tumor growth for hemopoietic cancers, prostate cancer, breast cancer, and renal cancer. The authors also report that somatic mtDNA mutations can be involved in breast, colorectal, bladder, esophageal, head and neck, ovarian, renal, lung and thyroid cancer, and leukemia and can influence cancer progression and metastasis. The effect of somatic mtDNA mutations on tumorigenesis depends on the functional and threshold effects of the mutation (57). Different human populations have different human mtDNA haplotypes, each with a unique fingerprint of mtDNA polymorphisms, passed on through the maternal germline. These haplotypes correlate to the geographic origin of the population. Certain human haplotypes carry a higher risk of developing a particular type of cancer or a neurodegenerative disease during their lifetime than others (8, 41, 58).

More than 50% of mtDNA mutations involved in carcinogenesis are located in the 22 mitochondrial tRNA genes (58). The most common mtDNA mutation is the single nucleotide polymorphism, 3243A > G, which alters leucine mt-tRNA and thus affecting translation of the 13 respiratory subunits, resulting in fewer mitochondrial subunits and impaired OXPHOS (59, 60). Individuals with 10–30% faulty copies of tRNA^Leu^ may develop maternally inherited diabetes and deafness. People with 50–90% faulty copies are likely to develop mitochondrial encephalomyopathy, lactic acidosis, and stroke-like episodes (MELAS) (50, 59–65). The tRNA^Leu^ mutation results in variable forms of mitochondrial RC deficiency in different patients. By far, the most common finding in MELAS is complex I (RC-I) deficiency, whereas some patients have combined deficiencies of RC-I, RC-III, and RC-IV (59, 66). Other mt-tRNA mutations that play a role in human disease are: tRNA^Lys^, which is associated with myoclonic epilepsy, tRNA^Ser^ with deafness, and tRNA^Ile^ with cardiomyopathies (51).

In addition to mutations affecting the respirasome and tRNAs, a recent review by Gopisetty and Thangarajan (67) summarizes possible roles for mutations in mitochondrial ribosomal small subunit genes (MRPS) in human disease. The authors describe the roles of 30 new MRPS as well as the effect of known MRPS mutations on different cancers and other diseases, including developmental and neurodegenerative diseases, mitochondriopathies, cardiovascular diseases, obesity, and inflammatory disorders. They further provide evidence of the role of MRPS18-2 in carcinogenesis as a potential oncogene. Differential expression of specific MRPS genes has been associated with breast cancer, cervical cancer, non-small cell lung cancer, thyroid tumors, invasive glioblastoma, Burkitt’s lymphoma, pediatric hyperdiploid acute lymphoblastic leukemias, testicular germ cell tumors, endometrial carcinoma, and head and neck squamous cell carcinoma (67). Expression levels in cancers were heterogeneous both within the same tumor type and between different cancers. For example, MRPL42 overexpression has been described for breast, carcinoid, liver, endometrial, melanoma, and ovarian cancers. Downregulation was seen in pancreatic, renal, and urothelial cancer. Expression profiles change in response to cisplatin chemotherapy treatment and radiation, indicating a potential role for MRPS genes in cellular responses cytotoxic drugs or serve as biomarkers. Single nucleotide polymorphisms in MRPS genes have also been linked to cancer risk [reviewed in Ref. (67)].

### Drivers and Timing of mtDNA Mutations

Until recently, the generally accepted view was that mtDNA mutations are generated by ROS-mediated oxidative damage (36, 41). Generation of ROS in the respiratory chain is inherently part of OXPHOS. ROS play an important part in several signaling processes and their levels are kept in check by antioxidant enzyme systems in the mitochondrial matrix and IMS. However, in situations where OXPHOS is compromised due to misshapen respiratory complexes resulting in increased leakage of electrons to oxygen, ROS levels may overwhelm the antioxidant defense system and damage nearby mtDNA (11, 12).

DeBalsi and colleagues propose that mistakes made by the mtDNA replication and repair machinery can also generate mtDNA mutations (68). Human cells contain 17 distinct human DNA polymerases, but only polymerase gamma (Pol-γ) functions in mtDNA replication and repair. Nuclear-encoded Pol-γ holoenzyme consists of a catalytic subunit and an accessory subunit [reviewed by DeBalsi et al. (68)]. Pol-γ replicates mtDNA with high fidelity due to nucleotide selectivity and proofreading ability with one mis-insertion in every 500,000 new base pairs (69). Over 300 Pol-γ mutations have been linked to human disease, some manifest in adulthood and these are associated with aging, such as various forms of progressive external ophthalmoplegia (PEO) and Parkinson’s disease (PD) [reviewed in Ref. (68)]. The importance of Pol-γ in limiting mtDNA mutations was demonstrated by homozygous, but not heterozygous, mutator mice with a proofreading-deficient Pol-γ developing several age-related conditions and having a shortened lifespan. They accumulated mtDNA mutations that were not caused by oxidative damage, as their antioxidant capacities were the same and the extent of oxidative damage was similar to wild-type mice. The mutator mice acquired somatic point mutations, large deletions and multiple linear deleted mtDNA fragments. Another n-mitoprotein involved in mtDNA replication is the mtDNA-specific helicase Twinkle, which unwinds mtDNA for synthesis by Pol-γ [reviewed in Ref. (70)]. Overexpression of Twinkle in transgenic mice led to increased mtDNA copy number and OXPHOS and several twinkle mutations are associated with mitochondrial myopathy (68). Both oxidative damage and faulty replication are likely to contribute to the total mtDNA mutational load of a cell and the contribution of each mutational driver is likely to change over time.

### Repair of Faulty mtDNA

For the most part, mtDNA repair pathways mirror those that occur in the nucleus with the same or similar proteins alternatively spliced and targeted to the mitochondria [reviewed by Kazak et al. (71)]. Mitochondria have a robust base excision repair (BER) system, which mainly fixes oxidative DNA damage of mtDNA physically associated with IMM. Single strand breaks are sensed by PARP-1 and repaired by the BER enzymes. There is also evidence of double strand break (DSB) repair, with alternatively spliced nuclear DSB repair proteins or mitochondrial homologs from the non-homologous end joining and homologous recombination pathways present in the mitochondrial matrix. Mismatch repair for replication errors is present in mitochondria but the proteins involved are distinct from those in nuclear mismatch repair (71). The various mtDNA repair pathways employ a myriad of proteins, all of which are nuclear encoded. Although there is a certain amount of redundancy, upregulation, downregulation, and/or point mutations in mtDNA repair proteins will affect mtDNA integrity. Cancer cells with a compromised mtDNA repair capability will accumulate more mtDNA mutations over time. In the event that mtDNA mutations are not removed through fission and mitophagy, increased mtDNA burden will compromise OXPHOS and force a switch to a purely glycolytic metabolism, as described in the next part of this review.

## Mito-Nuclear Cross Talk

**Figure 2:**
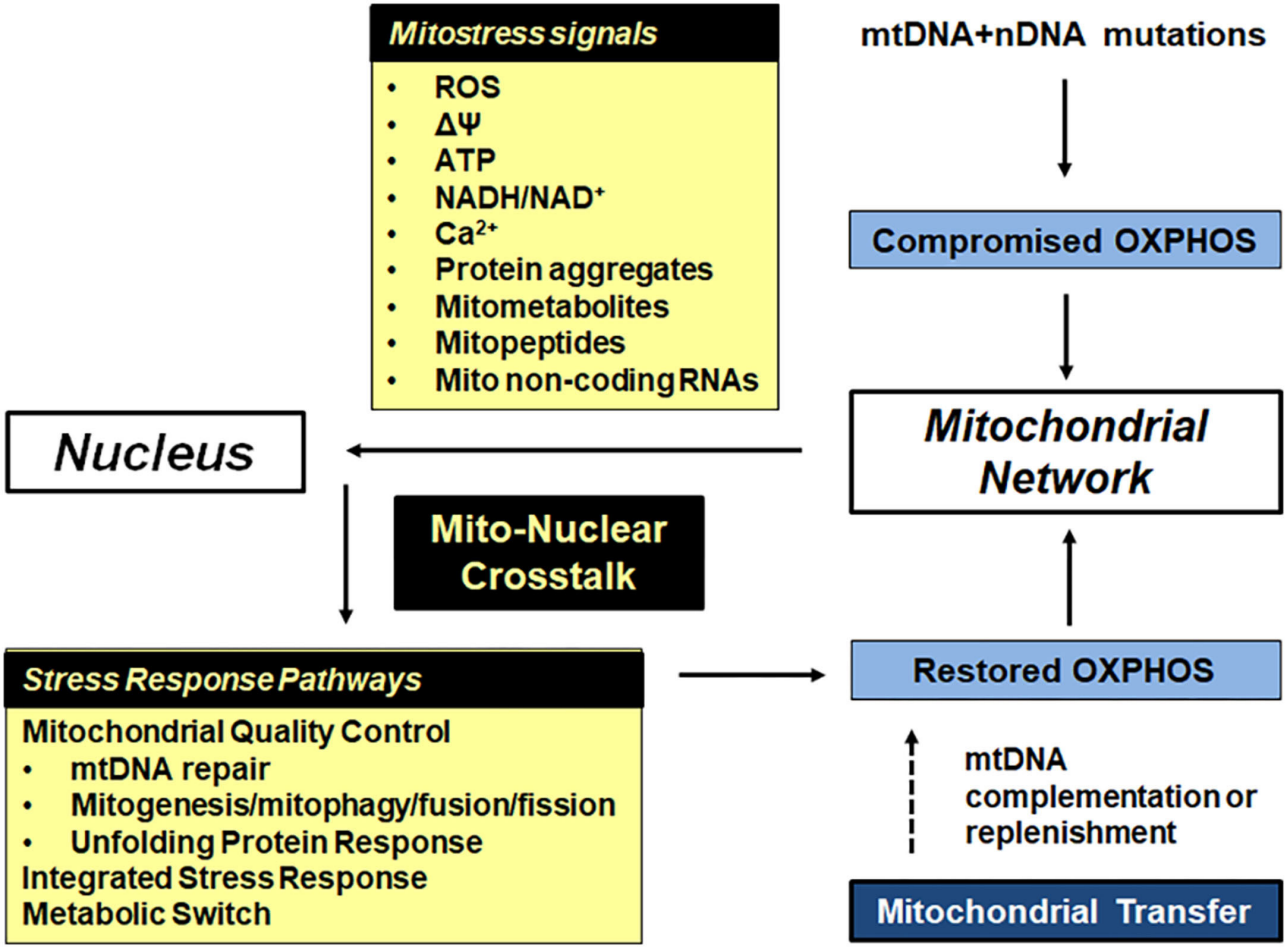
Bioenergetic plasticity is central to the ability of cells to adapt to their ever-changing microenvironment. Mitochondrial and nuclear mutations in respiratory subunits, mitochondrial chaperones and proteases and other mitochondrial proteins can lead to compromised oxidative phosphorylation (OXPHOS). In response, damaged mitochondria send out mitostress signals such as increased reactive oxygen species (ROS) and exhibit decreased inner mitochondrial membrane potential. The latter causes a drop in mitochondrial ATP levels, increased mitochondrial and cytoplasmic NADH, and increased cytoplasmic calcium. In addition, mtDNA and nDNA mutations as well as increased ROS cause aggregation of damaged and misfolded proteins in the mitochondrial matrix, leading to prototoxic stress. Mitometabolites affect epigenetic modifications. The nucleus responds to these mitostress signals by activating one or more stress response pathways, depending on the type and duration of the stress signal. Repair of damaged DNA, dilution of faulty mtDNA copies by fusion and mitogenesis, removal of faulty mtDNA copies by fission followed by mitophagy, removal of protein aggregates by the mitochondrial unfolded protein response (mtUPR), removal of faulty mitochondria by the integrated stress response (ISR) and a shift from mitochondrial to glycolytic/lactate metabolism all ensure that the energy demands of cells are met. If OXPHOS cannot be restored, failing mitochondria are removed though mitophagy. In extreme cases, the cell undergoes apoptosis or replaces faulty mitochondria with functional mitochondria from other cells.

Most mito-nuclear cross talk is focused on meeting the bioenergetics demands of cells. This will be related to the speed with which cellular demands change and the consequences if these demands are not met. Mitochondria continually update the nucleus of their bioenergetics status (retrograde signaling) by producing a number of energy metabolites (mitostress signals). The nucleus responds by activating stress response signaling pathways aimed at adjusting ATP production to suit the cell’s energy requirements. The different mitostress signals and nuclear stress response pathways are summarized in Figure 2.

### Mitostress Signaling Overview

Decreased MET results in a *decrease in mitochondrial membrane potential* which leads to several mitostress signals that trigger specific nuclear transcriptional responses described in more detail by Arnould et al. (72) and summarized in Figure 2. *Decreased mitochondrial ATP levels* cause energy deprivation (high AMP/ATP ratio). This induces AMP-activated protein kinase (AMPK) signaling, which activates peroxisome proliferator-activated receptor γ coactivator 1-α (PGC-1α), promoting mitogenesis. PGC-1α also decreases mammalian target of rapamycin (mTOR) activity which downregulates energy-demanding anabolic processes, which is mirrored by a lack of Krebs cycle metabolites available for anabolic pathways. *Increased cytosolic Ca^2+^levels* activate transcriptional regulators such as activating transcription factor, CREB1, NFkB, p53, MEF-2, and PGC-1α. *Increased mitochondrial NADH/NAD^+^ratios* affect the membrane and cytosolic redox potential, causing reductive stress. Changing the NADH/NAD^+^ ratio also affects activity of the NAD^+^-dependent poly[ADP-ribose] polymerase-1 (PARP-1), involved in DNA repair, and the Sirtuin family. *Increased mitochondrial ROS* result from leakiness of the respiratory chain caused by misshapen respiratory complexes. In most healthy mammalian cells, 95–98% of total oxygen consumption occurs at respiratory complex IV. Premature electron leakage to oxygen occurs at respiratory complexes I, II, and III generating superoxide, although other possible sites of superoxide production have been identified (73). Superoxide is converted to hydrogen peroxide by superoxide dismutases (Cu-ZnSOD in the IMS and MnSOD in the matrix). Hydrogen peroxide in mitochondria is detoxified to water and oxygen by glutathione peroxidase and peroxireductase (73). Under normal circumstances, these antioxidant enzymes neutralize most of the ROS, leaving enough hydrogen peroxide to pass through membranes to promote redox signaling through modifications of cysteine residues on redox sensitive proteins, resulting in posttranslational modifications. Redox signaling has been implicated in anti-aging and longevity, promoting protective stress responses and enhanced immunity [reviewed in Ref. (74)]. Excess hydrogen peroxide can be transformed into the highly aggressive hydroxyl radical, responsible for oxidative damage to mitochondrial proteins, lipid and DNA. Both SIRT1 and SIRT3 are activated under increased ROS and help orchestrate increased antioxidant gene expression as well as mitophagy.

#### Prototoxic Stress

Prototoxic stress is caused by a mismatch in the number/shape of respiratory chain subunits and results in a buildup of redundant/misshapen/unfolded respiratory subunits in the mitochondrial matrix. Both proteotoxic stress and depolarization of mitochondria activate the mitochondrial unfolding protein response (mtUPR), resulting in accumulation of PINK 1 in the IMS, recruitment of PINK2 to the mitochondria, and the removal of defective mitochondria through mitophagy (75).

#### Mitometabolite Levels

Mitometabolite levels such as acetyl-CoA and S-adenosyl methionine (SAM) affect acetylation and methylation of the nuclear genome, respectively. Changes in the nuclear and mtDNA profiles can directly affect epigenetic regulation and thus cancer progression and metastasis (76). SAM is the primary methyl donor molecule utilized in cellular methylation of proteins, DNA, RNA, and lipids and is synthesized directly from methionine. Both existing DNA, as well as newly synthesized DNA can be dynamically methylated and demethylated (76). In mouse embryonic stem cells, lack of threonine in the growth medium decreased accumulation of SAM and decreased histone methylation, resulting in slowed growth and increased differentiation (77).

### Mitopeptides

Recent mitochondrial transcriptome analysis have revealed the existence of several small open reading frames (sORFS) within the 16S and 12S rRNA gene sequences. These sORFS corresponded with small mitochondria-derived peptides (MDPs). The first MDP, humanin, was discovered in 2001 by Hashimoto et al. (78), followed by the discovery of MOTS-c by Lee et al. in 2015 (79) and small humanin-like peptides (SHLP 1–6) by Cobb et al. in 2016 (80). Emerging evidence suggests that MDPs play important roles in the regulation of cellular bioenergetics and system metabolism by modulating insulin sensitivity and glucose homeostasis [reviewed by Kim et al. (81)], but whether these hormone-like peptides are *bona fide* retrograde signaling molecules that modulate nuclear gene expression or induce epigenetic changes intracellularly or in other cells remains to be determined. The 24 amino acid humanin (located within the 16S rRNA gene) has been shown to be strongly neuroprotective, antiapoptotic, and protects against ischemia/reperfusion injury possibly due to a decrease in ROS generation (78). Humanin directly affects mitochondrial bioenergetics by increasing basal OCR, respiration capacity, and ATP production and increases mtDNA copy number and the number of mitochondria. Humanin also plays a role in lipid metabolism by decreasing body weight gain, visceral fat, and hepatic triglyceride accumulation together with an increase in activity level in high-fat diet-fed mice (81). Injecting humanin improved pancreatic islet function and insulin sensitivity in non-obese diabetic mice and prevented diabetic progression in some animals (82). In humans, humanin is found in the brain, hypothalamus, heart, vascular wall, blood plasma, kidneys, and testes (83). Humanin could be considered a mitohormone with plasma levels adjusting to cellular oxidative stress levels. In support of this notion, low levels of oxidative stress, as seen in prediabetic patients with slightly increased blood glucose levels, had significantly lower plasma humanin levels than healthy control patients (83). This could be considered a positive adaptation to mild-moderate oxidative stress which may promote longevity in a similar manner to dietary caloric restriction (84). As oxidative stress increases, mitochondria would then significantly upregulate humanin levels as seen in patients with advanced mitochondrial encephalopathy, lactic acidosis and stroke-like episodes (MELAS), and chronic progressive external ophthalmoplegia (85, 86).

The six SHLP peptide sequences (20–38 amino acids long) are also found within the 16S rRNA gene (80), with SHLPs1-5 being on the antisense light strand. SHLP2 and SHLP3 have similar protective effects as humanin and both improved mitochondrial metabolism by increasing oxygen consumption rate and ATP production, mitochondrial biogenesis and by reducing apoptosis and ROS levels. SHLP2 and SHLP3 enhance insulin sensitizing effects *in vitro* and *in vivo* (81). MOTS-c is a 16 amino acid peptide located within the 12S rRNA gene [reviewed in Ref. (87)]. MOTS-c increases glucose uptake and glycolysis through AMPK activation, whereas it suppresses mitochondrial respiration in cultured cells and skeletal muscle. This resembles a Crabtree effect-like phenomenon, namely, decreased mitochondrial OCR in response to high glucose uptake. MOTS-c is also closely associated with amino acid and lipid metabolism. MOTS-c enhances whole body insulin sensitivity, acting primarily through the muscle. MOTS-c further prevents HFD-induced obesity and insulin resistance in CD-1 mice and prevents HFD-induced obesity independent of caloric intake in C57BL/6J mice (87).

### Mitochondrial Non-Coding RNAs

Large parts of the non-coding nuclear genome, which itself represents more than 98% of the total genome, are transcribed into various types of non-coding RNA, which include rRNAs, tRNAs, small nucleolar RNAs, small nuclear RNAs, and the more recently identified microRNAs (miRNAs), and long non-coding RNAs (lncRNAs). According to the last GENCODE release (v25), the human genome contains more than 4,000 miRNA and 15,000 lncRNA genes. A very recent review by Vendramin and colleagues describes in detail the roles of different types of non-coding RNAs as modulators of mitochondrial function (88). The 22 nucleotide long miRNAs are highly conserved non-coding RNAs that have been implicated in a large variety of patho-physiological processes including aging and cancer. They inhibit translation of mRNA targets in the cytoplasm, by binding to them and recruiting the RNA-induced silencing complex. Many non-coding RNAs have evolved to allow cells to cope with stress and several miRNAS have been shown to play a role in tumorigenesis, both as oncogenes and tumor suppressors (88).

MicroRNAs have been found inside mitochondria of mtDNA competent cells while being absent in their mtDNA deficient ρ^0^ counterparts (89) These mito-miRNAs could have been nuclear or mitochondrially encoded and would have the potential to bind and prevent translation of mito-messenger RNAs. A decrease in the number of mitochondrially encoded respiratory subunits would affect respiratory subunit stoichiometry and thus mito-nuclear cross talk, resulting in cells switching to a more glycolytic metabolism, which is described in more detail below. Two mitochondrially encoded miRNAs were described very recently by Gao et al. after re-analyzing a public PacBio full-length transcriptome dataset, producing the full-length human mitochondrial transcriptome (90). The authors propose that these miRNAs, through sense–antisense interactions with mRNAs, regulate the transcription of the RC subunits, and thus control MET and OXPHOS activity. Interestingly, the transcription level of these miRNAs was significantly higher in normal tissues compared with hepatocellular carcinoma, indicating a loss of regulatory control (90).

### Metabolic Shift

Glycolysis is the common energy-generating pathway used by all mammalian cells; it oxidizes glucose to pyruvate in the cytoplasm, generating 2ATP/glucose through substrate phosphorylation. In the presence of oxygen, cells with a functional respiratory chain will further oxidize pyruvate to carbon dioxide in the Krebs cycle, generating 2ATP/glucose. Reoxidation of NADH and FADH_2_ during MET ideally generates an additional 30-32ATP/glucose through OXPHOS. Under hypoxic conditions, some normal cells (myocytes, hepatocytes, erythrocytes, and adipocytes) and most cancer cells reoxidize NADH produced during glycolysis *via* LDH that reduces pyruvate to lactate, and through a short evolutionarily conserved electron chain in the plasma membrane (PMET). PMET could be a potential evolutionary remnant of an ancient pathway responsible for preventing intracellular reductive stress due to buildup of NADH during glycolysis. A number of different PMET pathways have been described in yeasts, plants and mammalian cells [reviewed in Ref. (4)]. A PMET system, active in highly proliferative glycolytic cells (both non-transformed and cancer cells), uses oxygen as a terminal electron acceptor, reminiscent of MET (91, 92). Cell surface oxygen consumption has been reported for a number of cancer cell lines and can be 2–3× higher in cells devoid of mtDNA (ρ^0^ cells) (91–93). Cell surface oxygen consumption also contributes to the acidification seen in glycolytic cells, due to increased LDH activity resulting in increased lactate production (92). Cell surface oxygen consumption together with LDH activity are required for maintaining intracellular NADH/NAD^+^ balance of highly glycolytic cancer cells, and thus their invasive and metastatic potential. In support of this, inhibition of PMET by the external redox cycler, phenoxodiol, was shown to promote apoptosis in a range of cancer cell lines (94–96) as well as leukemic blasts from patients with myeloid and lymphoid leukemias (97, 98). Cell-impermeable drugs targeting PMET may, therefore, represent useful additional tool in preventing growth, invasion, and metastasis of highly glycolytic cancers (4, 99–101).

The metabolic shift from OXPHOS to aerobic glycolysis in rapidly proliferating cells, including cancer cells is controlled by hypoxia-inducible factor 1α which is highly expressed in most solid cancers [reviewed in Ref. (102)]. However, even under aerobic conditions, many, but not all, cancer cells rely to a large extent on glycolysis to meet their energy demands. This allows them to use glycolytic intermediates for anabolic processes and escape the effects of high ROS levels at the expense of additional OXPHOS energy. Otto Warburg was the first person to describe the phenomenon of aerobic glycolysis (the Warburg effect) in the 1920s in Ehrlich ascites cells (103). However, other than for cells unable to use OXPHOS due to an assortment of mutations, this scenario has proven to be too simplistic. Many cancer cells still use OXPHOS to increase their bioenergetic potential and generate low levels of ROS for signaling purposes. It seems that the glycolysis to OXPHOS shift is more like a rheostat, facilitating a dynamic adjustment of the proportion of energy gained from glycolysis and OXPHOS depending on demand and the microenvironment (20). For example, ionizing radiation causes re-oxygenation in previously hypoxic tumors. In this scenario, cancer cells with the flexibility to adjust readily between glycolysis and OXPHOS would have a distinct survival advantage. In support of this, Lu and colleagues recently showed that exposing human MCF-7 breast cancer cells, HCT116 colon cancer, and U87 brain cancer cells to a single dose of 5 Gy caused a switch from aerobic glycolysis to OXPHOS, increasing their bioenergetic capacity and conferring radiation resistance (104). They reported that mTOR, a serine/threonine kinase of the PIK3 family and highly expressed in cancer cells, translocated to the OMM after radiation. There, mTOR bound to and inactivated hexokinase II, inhibiting glycolysis and reactivating OXPHOS (104).

### Mitochondrial Quality Control

**Figure 3:**
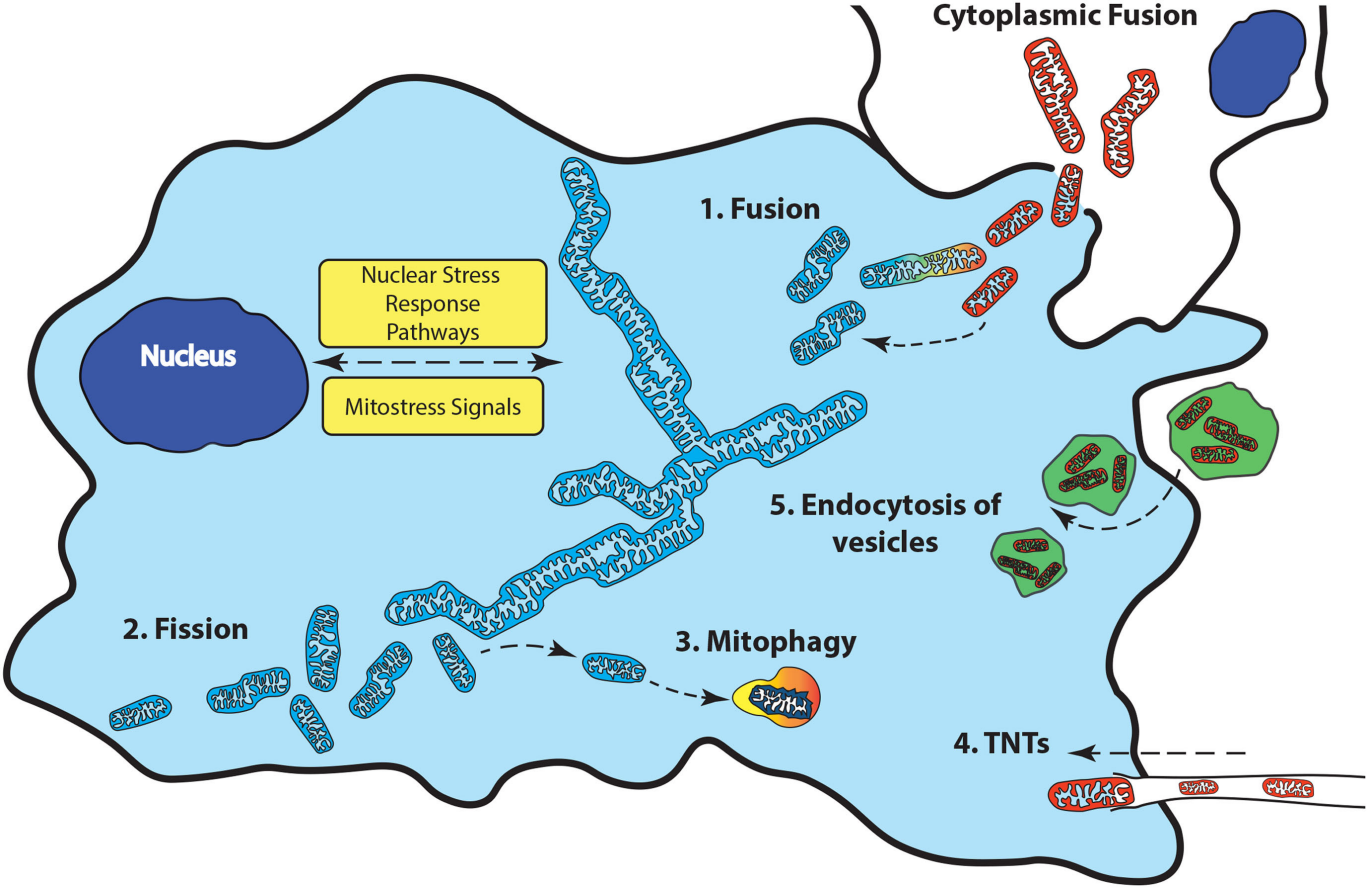
Mitochondrial quality control involves changes to the mitochondrial network to maintain bioenergetics homeostasis. Fusion (1) of additional mitochondria to the existing network occurs when the bioenergetics demands of the cell are not met. When damaged mitochondria cannot be repaired, they can be removed from the network through fission (2) followed by removal from the cell though mitophagy (3). Transfer of functional mitochondria from external sources through tunneling nanotubules (TNTS) (4), vesicles (5), or direct cytoplasmic fusion (6) can replenish a damaged or deficient mitochondrial network.

In general, a shortfall in ATP levels is caused by a lack of respiratory units, increased energy demands by the cell, or transient hypoxia. In these cases, mitochondrial quality control restores the bioenergetics capacity of the cell for differentiated function by increasing the mitochondrial network through mitogenesis. In contrast, increasing glycolysis and removing excess mitochondria through autophagy (mitophagy) favors rapid cell proliferation (see Figure 3).

#### Mitogenesis

Mitogenesis is regulated by mitochondrial and nuclear-encoded structural proteins as well as n-mitoproteins involved in mtDNA transcription, translation, and repair. Expression of the master regulator of mitochondrial biogenesis, PGC-1α is increased with increased energy demands after exercise in skeletal and cardiac muscle as well as upon fasting in the liver, or after cold exposure in brown fat cells. PGC-1α regulates the transcription levels of a number of genes involved in metabolic pathways such as gluconeogenesis, fatty acid synthesis, and oxidation, promoting mitochondrial biogenesis, angiogenesis, and aerobic respiration [reviewed in Ref. (105)].

Regulation of PGC-1α occurs at the level of expression and a variety of posttranslational changes that regulate its activity and stability (phosphorylation, acetylation, and ubiquitination). Activation of the p38 mitogen-activated protein kinase (p38 MAPK) increases PGC-1α expression and stability of the protein in brown fat, muscle and liver, which increases gluconeogenesis. The energy sensor AMPK also induces PGC-1α transcription and enhances its activation through Sirt1-mediated deacetylation when ATP/ADP levels are low (105). This in turn allows for the regulation of the downstream pathways controlled by PGC-1α. One of the targets of PGC-1α, is the newly discovered peptide hormone, irisin, secreted by muscle cells after exercise (106). Increased irisin expression caused browning of subcutaneous adipose tissue (thermogenesis), increased oxygen consumption, reduced obesity and insulin resistance in mice given a high fat diet (106).

The effects of PGC-1α in cancer cells mimic those in normal cells including mitochondrial biogenesis and increased OXPHOS, with the added effect of promoting invasion and metastasis (107). Circulating breast cancer cells have been found to exhibit enhanced mitochondrial biogenesis and respiration as a result of increased PGC-1α expression, leading to an increased rate of metastasis (107). PGC-1α also increased resistance to cisplatin of ascites-derived cancer cells from ovarian cancer patients with advanced disease (108). In addition, expression of PGC-1α and TFAM were increased in high grade serous ovarian cancers that were highly chemoresistant (109).

#### Mitophagy

Mitophagy is crucially important in removing superfluous or faulty mitochondria from the cell. Mitophagy is triggered by the PTEN-induced putative kinase 1 (PINK1)/Parkin pathway. This pathway is activated by membrane depolarization which is a signal of mitochondrial dysfunction caused by hypoxia, lack of NADH, and/or a limited number or ill-fitting dysfunctional respiratory complexes. Stabilization of PINK1 on the depolarized OMM directly phosphorylates Parkin, which ubiquinates a number of OMM proteins leading to their degradation through the 26S proteasome and recruitment of the autophagosome. Other Parkin-dependent and independent mitophagy pathways have been described and have been reviewed in detail by Gumeni and Trougakos (110).

Faulty mtDNA copies can be diluted out through continued cycles of fusion/fission events. Fusion of several individual mitochondria into the larger network allows for complementation of mtDNA variants to maintain mitochondrial function. Parts of the mitochondrial network with a high mutational load can be isolated through fission and eliminated through mitophagy (111, 112). *Fusion* is orchestrated by three GTPases; the mitofusins, Mfn1, and Mfn2, are involved in fusion of the OMM, whereas optic atrophy-1 (Opa1) is responsible for fusing the IMM and is also involved in cristae remodeling (111–113). *Fission* is driven by recruitment of dynamin-related protein 1 (Drp1) to receptors on the OMM where it causes constriction of both the OMM and IMM (1). Drp1 translocation and activity is regulated by multiple kinases that respond to distinct cell cycle and stress conditions (113).

### Removal of Protein Aggregates by mtUPR

Accumulation of ROS-damaged/unfolded/misfolded proteins in the mitochondrial matrix is called prototoxic stress. The mitochondrial unfolded protein response (mtUPR) is responsible for degrading protein aggregates in the mitochondrial matrix and IMS. Two comprehensive reviews describe the process of unfolding, translocation, and refolding of precursor proteins, as well as degradation of damaged/misfolded/unfolded proteins by ATP-dependent and ATP-independent proteases and oligopeptidases (72, 110). Most of our knowledge of the mtUPR pathway and its integration with other stress pathways has been obtained from research with *C. elegans*. The mtUPR in mammals is still poorly defined with respect to signaling pathways and target genes. Two separate mtUPR pathways have been described, one deals with protein aggregates in the mitochondrial matrix and the other resolves protein aggregates in the mitochondrial IMS (72, 110). An interesting recent review by Nuebel and colleagues explores the roles of many newly identified proteins unique to the IMS in mitochondrial and cellular homeostasis (114). This mitochondrial compartment represents a uniquely oxidizing redox environment. The IMS proteome is involved in protein and lipid transport across the OMM and IMM, apoptosis, redox homeostasis, ROS signaling, and MET. Accumulation of unfolded or aggregated proteins is a hallmark of neurodegenerative diseases such as Alzheimer’s disease and Parkinson’s disease. Prototoxic stress in the mitochondrial matrix is also a common occurrence in cancer cells and many, but by no means all cancer types, have an activated mtUPR response (110).

### Integrated Stress Response (ISR)

The ISR is an evolutionary conserved adaptive stress pathway. In mammals, the ISR is activated under oxidative stress, ER stress, depletion of amino acids, glucose and haem, viral infection, or UV irradiation. Four different kinases phosphorylate the eukaryotic translation initiation factor 2α (eIF2α), a key event in ISR [described in detail in Ref. (72)]. Activation of eIF2α reduces ATP-consuming processes such as protein synthesis, helps stabilize Ca^2+^ storage in the ER and mitochondria, and maintains mitochondrial function. If mitochondrial function cannot be recovered, the ISR can initiate autophagy or apoptosis. Mitochondrial ISR is triggered by mitochondrial dysfunction caused by mtDNA damage, mtDNA depletion, and oxidative stress, whereas mtUPR is specifically triggered by protein aggregates buildup in the mitochondrial matrix. However, it is likely that these two stress pathways overlap as phosphorylation of eIF2α is a common feature of both pathways.

## Mitochondrial Transfer Between Cells

So far, this review has covered the many different ways that cells address inadequate mitochondrial performance. However, what happens when mito-nuclear cross talk fails, mtDNA and nDNA mutations that affect mitochondrial function accumulate, ATP levels fall and biosynthetic pathways wind down? Until fairly recently, the answer to these questions would have been clear: a decrease in metabolic rate and ultimately, cell death would ensue. However, recent research has shown that cells may be able to obtain functional mitochondria from other cells in order to satisfy their bioenergetics and biosynthetic needs.

The traditional cell biology dogma that mitochondria and mtDNA remain within the constraints of their host cell has recently been questioned. Several studies have shown that mitochondria can move between cells *in vitro* (115–124). Furthermore, we recently showed that tumorigenesis of murine melanoma and breast cancer cell lines without mtDNA depended on their ability to obtain mtDNA from host mouse cells in the microenvironment (125, 126).

Transfer of functional mitochondria was also shown to confer a survival advantage in several mouse models. For example, bone marrow-derived stromal cells were able to rescue lipopolysaccharide-induced acute lung injury in alveolar epithelia of mice (127) while transfer of mitochondria from mesenchymal stem cells (MSCs) protected epithelia by decreasing mitochondrial ROS in a mouse model of airway injury and allergic airway inflammation (128). Transfer was enhanced when the donor cells overexpressed Miro1, a mitochondrial Rho-GTPase. In other recent publications, astrocytes were shown to increase ATP levels and viability of neurons in a mouse ischemia model by donating healthy mitochondria contained in vesicles (129), while stromal cells transferred mitochondria to immortalized acute myeloid leukemia (AML) cells in an immunocompromised mouse xenograft model in response to chemotherapy-induced apoptosis (130). This transfer occurred *via* endocytosis from stromal cells to the AML cells, and increased ATP production, viability, and survival of AML cells was reported. Mitochondrial transfer has also been shown to rescue aerobic respiration in carcinoma cells (116) and increase survival in an adrenal gland cell line (117). Together, these results suggest that intercellular mitochondrial transfer plays a role in cellular communications, intracellular metabolic homeostasis or exists as a mechanism to support cells under physiological stress. In support of this, recent research demonstrated that cells exposed to injury have improved survival when introduced to healthy cells as an extracellular source of mitochondria (115, 127, 128, 131). When exposed to intentional injury such as chemotherapy or radiation, fragmentation of mtDNA occurs alongside damage to the nuclear genome. The resulting mitochondrial dysfunction in the absence of nuclear DNA damage can be toxic (132) and/or contribute to the mechanism of action of several cancer therapies. Circumvention of mtDNA damage by uptake of mitochondria from other cells could lead to treatment resistance.

Although most published work refers to mitochondrial transfer as a way to replace dysfunctional mitochondria, cells could also transfer dysfunctional mitochondria (133, 134). This may be particularly relevant in both Alzheimer’s and Parkinson’s disease where an increase in mitochondrial dysfunction is correlated with progressive degenerative phenotypes (111–113). Mutations in mtDNA and disrupted mitochondrial homeostasis are common across many neurodegenerative diseases and lead to mitochondrial dysfunction (114). Affected cells could manage the increase in faulty mtDNA copies by intercellular transfer of dysfunctional mitochondria that escape mitophagy.

The types of cells that are able to donate mitochondria, as well as the communication between recipients and donor cells that drive this transfer remain largely unknown. The exact mechanism(s) of mitochondrial transfer has also not been fully elucidated. Tunneling nanotubes (TNTs), extracellular vesicles and direct cellular contact have all been suggested to facilitate mitochondrial transfer between cells (see Figure 3).

### Tunneling Nanotubes

Emerging research into the role of TNTs has revealed that these open-ended, F-actin containing intercellular structures can act as a conduit for intercellular transfer of various biomaterials, inclusive of, but not limited to mitochondria (115, 131, 135–138). First described in 2004 (139), movement of organelles through TNTs has received much attention, particularly from investigators engaged in MSC research. Numerous recent studies conclude that intercellular mitochondrial transfer contributes to the protective or restorative properties of MSC seen both *in vitro* and in multiple animal injury models [reviewed in Ref. (140)]. The formation of TNT-like structures is closely associated with the physiological state of the cells, making TNTs likely candidates for facilitating intercellular mitochondrial transfer *in vivo* (133, 141–143). However, due to the challenges in identifying and characterizing open-ended TNTs among a multitude of other subcellular tube-like structures, definitive evidence in support of TNT-mediated mitochondrial transfer *in vivo* remains elusive (144). Identification of TNTs is currently limited to generalized morpho-temporal characteristics determined by time-lapse confocal fluorescence microscopy *in vitro* (129). Identification of specific markers for TNTs and other similar structures engaged in intercellular mitochondrial transfer is a prerequisite for further progress in the field.

### Extracellular Vesicles

Microvesicles, exosomes, apoptotic bodies, and oncosomes are biological particles which fall under the broad categorization of “extracellular vesicles.” Vesicle size, molecular content, and the origin of the particles determines their specific nature and biological role [reviewed in Ref. (145)]. Microvesicles make up the largest category of extracellular vesicles and consist of particles of up to approximately 1 µm in size. Like other extracellular vesicles, microvesicles bear proteomic signatures that allow cellular uptake *via* endocytosis or phagocytic mechanisms. Their molecular contents can exert a broad range of effects on cell physiology. Numerous studies of intercellular mitochondrial transfer report mtDNA as well as intact mitochondria can be partitioned into microvesicles from specific cell types, suggesting a vesicular mechanism for uptake of exogenous mitochondria by recipient cells. The first published study of intercellular mitochondrial transfer by Spees et al. (115) reported secretion of extracellular vesicles containing mitochondrially targeted fluorescent proteins by human mesenchymal stem cells into growth medium and uptake by recipient cells. These particles were also found to play a role in intercellular transfer of mitochondria by Islam et al. (127) who observed connexin43-mediated uptake of bone marrow-derived MSC mitochondria in microvesicles by lung epithelium. Evidence for mitochondrial and mtDNA transfer mediated by extracellular vesicles has seen steady development across many different cell types, and continues to expand as a new field of intercellular communications (129, 133, 141, 143, 144).

### Partial or Complete Cell Fusion

Perhaps the least explored mechanism in the existing mitochondrial transfer literature, the acquisition of exogenous mitochondria *via* partial or complete cell fusion, is an interesting concept. Given the notion that the majority of cells exist in a state of individual compartmentalization, cells engaged in these types of intimate interactions may not be limited to traditional syncytial candidates such as osteoclasts or skeletal muscle cells. Cell fusion may be more widespread and important within normal biological function than traditionally thought—this is a somewhat challenging proposition. There is precedence for certain cell types, particularly those derived from the bone marrow, to spontaneously fuse with other cell types including cardiomyocytes, hepatocytes, and Purkinje neurons (146–150). Partial fusion events or alternatively, syncytial mixing through intercellular structures, provides opportunities for the acquisition of mitochondria from surrounding cells. An example of this would be the tumor networks interconnected by tumor microtubes (distinct from TNTs) in primary glioblastomas, that communicate *via* connexin43 gap junctions (151, 152). Spees and colleagues (115) demonstrated mitochondrial transfer without the uptake of nuclear associated polymorphisms, excluding complete cell fusion in their system. Regardless, restoration of bioenergetic status and cellular regeneration *via* fusion-like mechanisms (150, 153) remains a potential mechanism in future studies of intercellular mitochondrial transfer.

## Concluding Remarks

**Figure 4:**
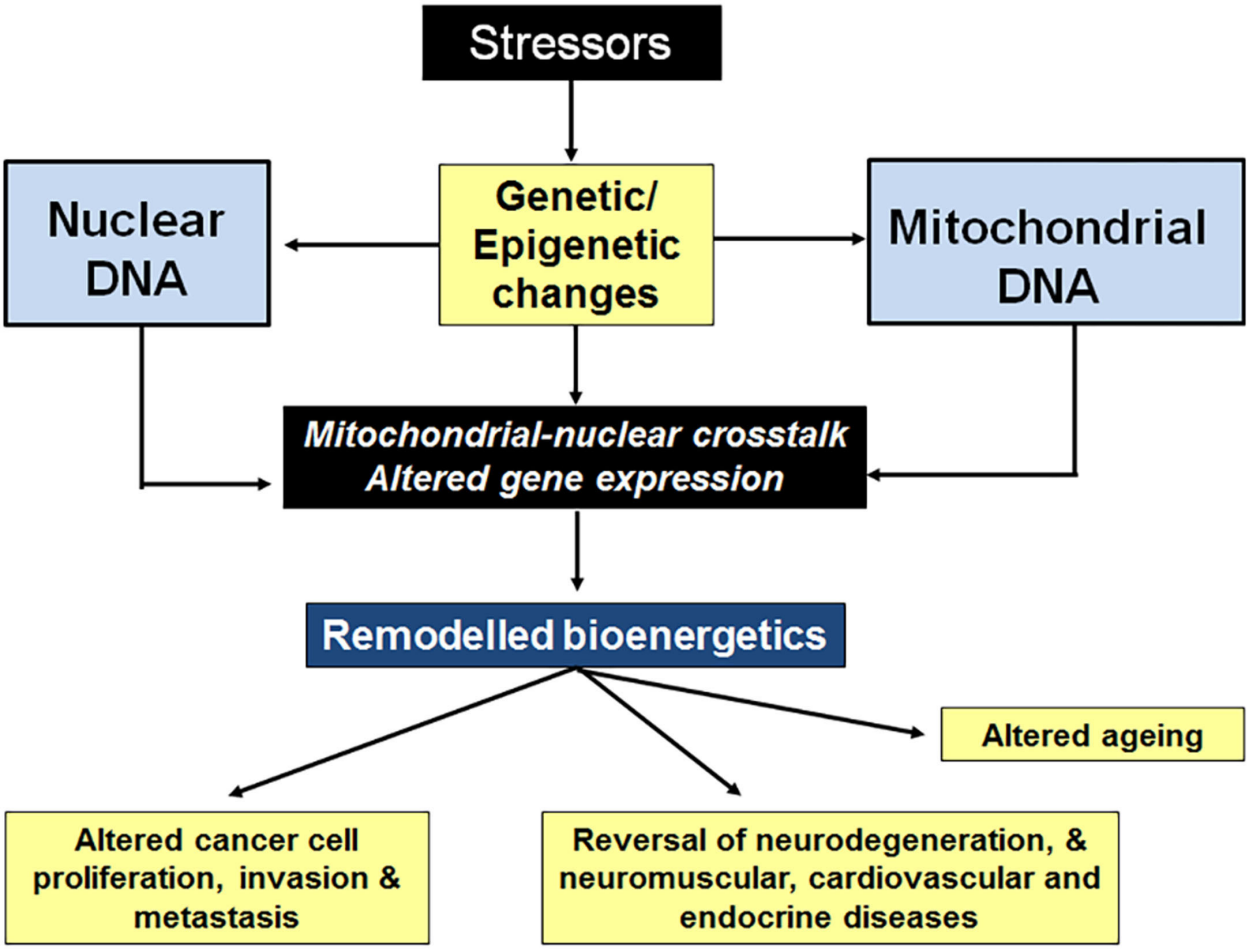
Stressors affect nuclear cross talk through changes in gene expression. Stressors such as oxidative stress, radiation, chemotherapeutic drugs, hypoxia, etc., cause genetic and epigenetic changes to both nDNA and mitochondrial DNA (mtDNA). The resulting changes in gene expression result in altered cellular bioenergetics, often leading to decreased oxidative phosphorylation. Mitochondrial stressors (a decrease in mitochondrial membrane potential, ATP levels, NADH levels, and increased mitopeptide expression, etc.) elicit nuclear stress responses. Stress pathway activation (mtDNA damage repair, mitochondrial biogenesis and fusion, switching to glycolytic metabolism, etc.) results in a return to bioenergetics homeostasis, restoring cellular function.

The ability to adapt cellular bioenergetics capabilities to meet rapidly changing environmental conditions is mandatory for cellular function and for cancer progression. Any compromise in this adaptive response has the potential to compromise cellular function and render the cell more susceptible to external stressors such as oxidative stress, radiation, chemotherapeutic drugs, hypoxia, etc. Mito-nuclear cross talk, involving the generation of different mitochondrial stressors as well as the nuclear stress response pathways to deal with those stressors is capable of maintaining bioenergetics homeostasis under most conditions (see Figure 4). Although many mito-nuclear stress signaling pathways have been described (see Mito-Nuclear Cross Talk), a detailed understanding of how these pathways work together to ensure that mitochondrial and nuclear transcription are closely coordinated to meet the dynamic bioenergetic and metabolic demands of the cell remain poorly understood. For example, the way in which the 13 mitochondrially encoded proteins of the mitochondrial RC that are made in mitochondria are combined with 80 nuclear-encoded proteins that are translated on distinct cytoplasmic protein synthetic machinery and imported into mitochondria where they are assembled into functional RCs is not fully understood. In addition, the role of the eight mito-peptides encoded by mitochondrial *rRNA* genes in bioenergetics and metabolic regulation is under intense scrutiny. Whether or not mitopeptides, or the lncRNA molecules transcribed from the light chain of mtDNA play a role in intracellular mito-nuclear cross talk is not known. The existence of many serious diseases caused by mitochondrial dysfunction, such as the neuromuscular and neurodegenerative mitochondriopathies (47–49), diabetes (50), cardiovascular diseases (51, 52), gastrointestinal disorders (53), skin disorders (54), aging (55, 56), and cancer (41), shows that mito-nuclear cross talk can fail. The ability to replace a dysfunctional mitochondrial network with fresh functional mitochondria from healthy cells is a recently discovered and currently poorly understood phenomenon. Mitochondrial transfer poses a series of intriguing questions: is mitochondrial transfer between cells a fundamental physiological process, silent until recently because of limited tools available for tracking mitochondrial movement between cells? If mito-nuclear cross talk is fundamental to cellular bioenergetic homeostasis, how does this process change when the original nucleus is faced with mitochondria from different cell types and/or with different genetic backgrounds following transplantation? Are the signals that promote mitochondrial transfer between cells related to those that are involved in mito-nuclear cross talk? Is this a process of trial error that takes time to be perfected?

On another front, comparative transcriptome analysis of both protein coding and non-coding mitochondrial and nuclear genomes, under various conditions could provide unbiased information about the immediate consequences of mito-nuclear cross talk that result in respiration and metabolic remodeling. Such analyses are likely to reveal unexpected regulatory roles for transcripts that could challenge current dogma about the stoichiometry and function of mitochondrial- and nuclear-encoded RC subunits, and the control of respiration. Large datasets often contain both mitochondrial and nuclear transcript information but these have rarely been mined for their comparative transcript information.

Finally, we are optimistic about the potential to look beyond current technological and conceptual horizons to better understand how bioenergetic and metabolic remodeling play out in health and disease. Cancer cells display enhanced plasticity with respect to metabolic remodeling at different stages of initiation, invasion, and metastasis, and in response to the many stressors they encounter in their rapidly changing microenvironment.

## Author Contributions

Concept of the review: MB; design of the review: MB and PH. PH wrote the review with contributions from MB, MR, and GC; PH made Figures 1, 3 and 4; MR made Figure 2. All authors were involved with finalizing the manuscript and all approved the final version.

## Conflict of Interest Statement

The authors declare that the research was conducted in the absence of any commercial or financial relationships that could be construed as a potential conflict of interest.
